# First record of Jacobsoniidae (Coleoptera) from China with description of a new species of *Sarothrias* Grouvelle

**DOI:** 10.3897/zookeys.496.8620

**Published:** 2015-04-16

**Authors:** Wen-Xuan Bi, Chang-Chin Chen, Mei-Ying Lin

**Affiliations:** 1 Key Laboratory of Zoological Systematics and Evolution, Institute of Zoology, Chinese Academy of Sciences, Beichen West Road, Chaoyang, Beijing, 100101, China; 2 Room 401, No. 2, Lane 155, Lianhua South Road, Shanghai, 201100, China; 3 NPS office, Tianjin New Wei San Industrial Company, Limited, China

**Keywords:** Jacobsoniidae, *Sarothrias*, new record, new species, taxonomy, Oriental region

## Abstract

The family Jacobsoniidae Heller (短跗甲科) is newly recorded from China upon the discovery of *Sarothrias sinicus* Bi & Chen, **sp. n.** (中华短跗甲) from Motuo, Southeast Xizang. Description and illustrations of the habitus and major diagnostic features of the new taxon are provided. A key to the species of *Sarothrias* and some ecological notes on the new species are presented.

## Introduction

The family Jacobsoniidae Heller, 1926 was considered *incertae sedis* within the series Bostrichiformia by Löbl and Burckhardt (1988), Lawrence and Newton (1995) and Lawrence et al. (1999a, b). Lawrence et al. (2010) provisionally placed Jacobsoniidae within the series Derodontiformia, along with families Derodontidae and Nosodendridae. In the current study, the results of a cladistic analysis based on morphological characters of adults and larvae indicated that Jacobsoniidae is sister to part of the Staphylinoidea (Lawrence et al. 2011).

Jacobsoniidae includes 21 known species in three genera: *Sarothrias* Grouvelle, 1918, *Saphophagus* Sharp, 1886 and *Derolathrus* Sharp in Sharp & Scott, 1908 (Háva and Löbl 2005). They can be recognized by the minute (0.65–2.5 mm), narrowly elongate (about 2.1–3 times as long as wide), yellowish brown body, an elongate prothorax, lack of a visible scutellum, and markedly elongate metaventrite (at least 2.5 times as long as mesoventrite). All species are poorly represented in collections and little is known about their biology (Lawrence and Leschen 2010; Philips et al. 2002).

Currently, 13 species of *Sarothrias* have been described (Háva and Löbl 2005; Pal 1998). They are restricted to humid tropical areas which close to the equator with the exception of *Sarothrias hygrophilus* Pal, 1998 from northeast India. They can be primarily defined and separated from *Saphophagus* by tarsal formula 3-3-3 instead of 5-5-5, and from *Derolathrus* by antennal club 3-segmented instead of 1- or 2-segmented (Burckhardt and Löbl 1990; Lawrence and Leschen 2010).

During July to August 2014, the first author participated in an expedition to Motuo (=Mêdog), Xizang, the third time that he visited that area. A small but remarkable beetle collected during this expedition represented an interesting, unexpected result in that it is an undescribed species of *Sarothrias* which belongs to the Jacobsoniidae – a family so far not recorded from China. In this paper, we describe a new species, *Sarothrias sinicus* Bi & Chen, sp. n., based on this specimen. An identification key to the species of *Sarothrias* is also included. Based on Lin and Yang (2012), Jacobsoniidae is the 147^th^ family of Coleoptera recorded from China.

## Results

### Sarothrias sinicus

Taxon classificationAnimaliaColeopteraJacobsoniidae

Bi & Chen sp. n.

http://zoobank.org/69D14BBA-16B8-49AC-8E45-AA254FB6C862

Figs 1–4 , 5

#### Type material.

**Holotype:** male, “China: Xizang, Motuo / Baricun / 2014.VII.27 1850 m / leg. Wen-Xuan Bi” [white label printed]. The holotype was deposited in the Insect Collection of Shanghai Normal University, Shanghai, China (SNUC).

#### Diagnosis.

This new species can be separated from most congeners by the elytra, which are predominantly shiny, and elytral row 5 unimpressed; the pronotum is devoid of squamiform setae. It differs from *Sarothrias hygrophilus* Pal by the elytra, with row 1 largely represented by fine punctures, row 2 interrupted by punctures after basal quarter, and with supplementary series (s1) between rows 3 and 4; the pronotum has secretions on the lateral margins instead of on the side below the protrusions. It also resembles *Sarothrias papuanus* Ślipiński but can be easily distinguished by the elytra, bearing four squamiform setae in the distal half of row 2, row 3 merging with row 2 and with s1 between rows 3 and 4; the pronotum is devoid of secretions.

#### Description.

**Figures 1–4.**
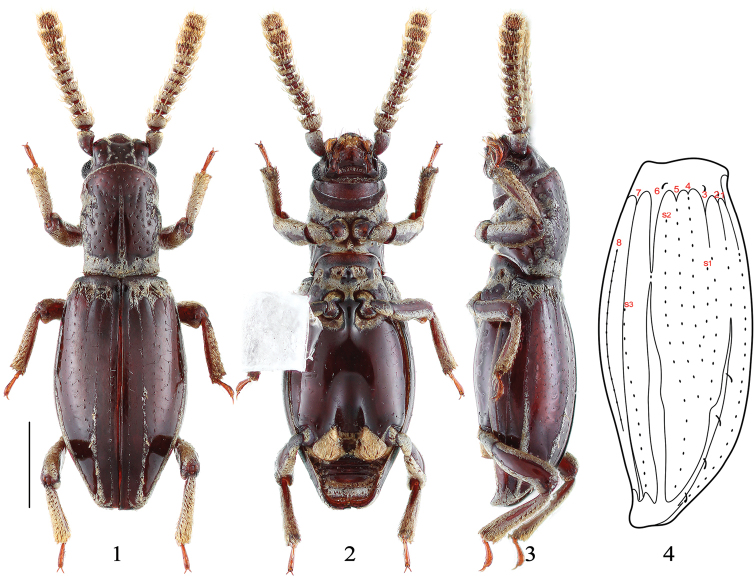
Habitus of *Sarothrias sinicus* Bi & Chen, sp. n., Holotype, male. **1** dorsal view **2** ventral view **3** lateral view **4** left elytron in three-quarter view (s1 to s3: supplementary series, s2 = s in Poggi 1991). Scale bar: 0.5 mm (**4** not to scale).

**Male** (Figs 1–3). Body length 2.20 mm, elongate, convex, dark-brown, dorsal surface largely shiny except whitish secretions in parts of pronotum and elytra; legs, excluded tarsi, pubescent and covered with yellowish secretions except two narrow longitudinal shiny band at both sides of femora and tibiae, tarsi brown; wings fully developed.

Head broader than long, width across eyes 0.45 mm, clypeus smooth, rounded anteriorly, fronto-clypeal suture clearly visible; frons slightly broad anteriorly with sides moderately raised, sparsely and distinctly setigerous punctured; eyes large, rounded, nearly as long as half of head length, coarsely facetted. Antenna length 0.88 mm, scape slightly elongate, antennomeres 2–11 transverse, with secretions on antennomeres 1–8, squamiform setae at apex of scape and on antennomeres 2–10 and normal setae on antennomeres 9–11.

Pronotum length 0.67 mm, width 0.48 mm, subparallel-sided, widest near middle; anterior margin rounded; disc with a shallow median groove, extending from the anterior one-fifth to little above base, slightly broad posteriorly; punctures on disc of similar size, shape and distribution to those on head; median groove, lateral margins and one-sixth of pronotal base with secretions. Scutellum invisible.

Elytra length 1.38 mm, width 0.79 mm, fusiform, widest slightly after middle; basal transverse bulge well developed, with subbasal band of secretions on which three subbasal depressions on each elytron, each depression with one squamiform setae posteriorly. Each elytron with striae or fine puncture forming 8 rows and 3 supplementary series (Fig. 4) of which 6 rows and 2 supplementary series are visible in dorsal view; rows 1 to 3 impressed at basal quarter and continued as a row of fine punctures, row 2 once again impressed after middle and connected with row 1 subapically, row 3 joined row 2 at apical one-third, rows 4 and 5 represented by fine punctures and disappearing anterior to apical one-third, row 6 largely impressed but intercepted by single puncture at basal one-third, rows 7 and 8 impressed, of which the former extending subapically and the latter starting at basal quarter and extending half of elytra length; three supplementary series (s1, s2, s3) represented by fine punctures, of which s1 present between rows 3 and 4 which starting at basal quarter and joined row 4 anterior to basal half, s2 (=s in Poggi 1991) present between rows 5 and 6 which starting at basal one-sixth and ending subapically, s3 separate from row 7 at basal two-fifths and ending subapically between row 6 and 7; apical half of row 2 and entire length of row 6 with secretions, of which the former secretionary row with four squamiform setae asymmetrically arranged.

Prosternum and mesoventrite largely covered with secretions. Metaventrite covered with the same secretions after mesocoxal insertions (cavities) and between metacoxal insertions, other parts shiny with very sparser and fine punctures; with deep median impression in apical half which with rounded sides and not delimited by lateral ridges. Legs moderately long, tarsi 3-segmented.

#### Ecological notes.

**Figure 5.**
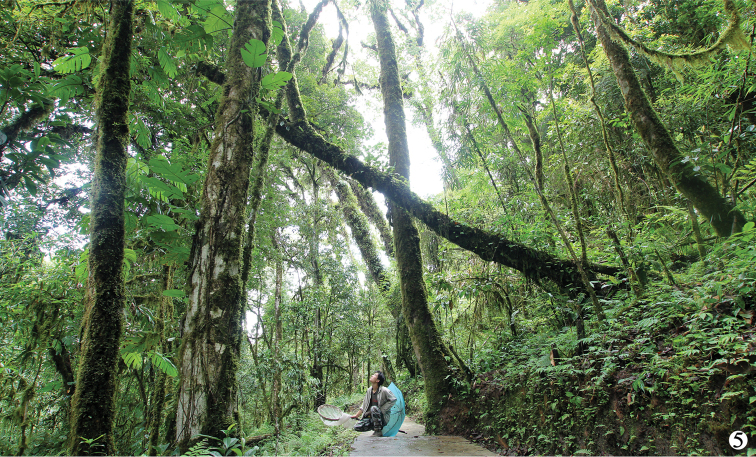
Habitat of *Sarothrias sinicus* Bi & Chen, sp. n., taken on the way from Baricun to Renqinbeng, Motuo, Xizang, alt. 1850 m.

Adult *Sarothrias* have been collected in leaf litter and rotten wood, but the larvae are as yet unknown (Lawrence and Leschen 2010). The only specimen of the new species was collected by beating a branch with dead leaves on the way from Baricun (= Bari village) to Renqinbeng (= Renqinbeng Temple), Motuo, Xizang at altitude of 1850 m (Fig. 5). The vegetation types are subtropical evergreen broadleaf forest which has been well protected, although slightly disturbed by locals by grazing. A variety of staphylinids, endomychids and cerambycids were collected at the same time. Another mysterious termitophilous lucanid, *Penichrolucanus cryptonychus* (Zhang, 1988), which is located in the same area and known only from its original description shares a similar generic distribution with *Sarothrias*.

#### Distribution.

China: Xizang (Tibet) Autonomous Region, Motuo County.

#### Etymology.

The new species is named after the country of the type locality.

### Key to the species of *Sarothrias*

(modified from Pal 1998)

**Table:** 

1	Elytra entirely mat and covered by secretions, except a narrow shiny stripe along the suture and which is devoid of secretions	**2**
–	Elytra partly shiny, not completely covered by secretions	**6**
2	Terminal antennomere with a whorl of squamiform setae as on antennomeres 2 to 10	**3**
–	Terminal antennomere devoid of a whorl of squamiform setae	**4**
3	Sides of pronotum with 2–3 squamiform setae; antennomere 2 twice as long as antennomere 3. Sabah	***Sarothrias crowsoni* Löbl & Burckhardt, 1988**
–	Sides of pronotum devoid of setae; antennomere 2 about 1.4× as long as antennomere 3. Moluccas	***Sarothrias audax* Ślipiński & Löbl, 1995**
4	Epipleural keel of elytra extending more towards base than lateral keel	**5**
–	Epipleural and lateral keels of elytra ending at about the same level. New Britain	***Sarothrias boumei* Ślipiński, 1986**
5	Median depression of metaventrite indistinctly delimited, narrowing towards apex beyond middle; mat, covered by secretions. Seychelles	***Sarothrias eximius* Grouvelle, 1918**
–	Median depression on metaventrite well delimited laterally, gradually narrowing towards apex; apical portion shiny, devoid of secretions. Fiji	***Sarothrias fijianus* Löbl & Burckhardt, 1988**
6	Secretions on head and pronotum strongly expanded, those on elytra forming longitudinal stripes which are separated by shiny stripes. South India	***Sarothrias indicus* Dajoz, 1978**
–	Dorsal surface of body predominantly shiny; pattern formed by secretions on elytra different	**7**
7	Pronotum with 2–3 squamiform setae on sides	**8**
–	Pronotum devoid of squamiform setae	**9**
8	Elytral secretions forming apical drop-shaped loop; antennomere 11 with squamiform setae. Sumatra	***Sarothrias dimerus* (Heller, 1926)**
–	Elytral secretions strongly reduced; antennomere 11 without squamiform setae. New Caledonia	***Sarothrias pacificus* Ślipiński & Löbl, 1995**
9	Elytron with row 5 entirely deeply impressed, merged. with row 4 apically. Queensland	***Sarothrias lawrencei* Löbl & Burckhardt, 1988**
–	Elytron with row 5 at most impressed near base and then continued as separate punctures, not joined with row 4	**10**
10	Elytron with rows 1 and 3 impressed only at base and then continued as a row of separate punctures	**11**
–	Elytron with rows 1 and 3 well impressed, row 3 with impression at least surpassing middle of elytron	**12**
11	Elytron with row 2 presented two squamiform setae apically, disconnected with row 3; devoid of s1. Papua New Guinea	***Sarothrias papuanus* Ślipiński, 1986**
–	Elytron with row 2 presented four squamiform setae after middle, connected with row 3; with s1 between rows 3 and 4 (Fig. 4). Southwest China	***Sarothrias sinicus* sp. n.**
12	Rows 2 and 3 of elytron entirely impressed, join far before level of metacoxae and at level of last puncture of row 5. Malaysia	***Sarothrias amabilis* Ślipiński & Löbl, 1995**
–	Rows 2 and 3 of elytron join near the level of metacoxae or behind it, further below level of last puncture of row 5, row 3 completely or incompletely impressed	**13**
13	Row 3 of elytron entirely impressed before joining with row 2. New Guinea	***Sarothrias morokanus* Poggi, 1991**
–	Row 3 of elytron impressed but interrupted just before joining with row 2, where it is represented by separate punctures. Northeast India	***Sarothrias hygrophilus* Pal, 1998**

## Supplementary Material

XML Treatment for Sarothrias sinicus

## References

[B1] Burckhardt D Löbl I (1990) Redescription of *Jacobsonium dimerum* Heller 1926, member of the rare tropical Indo-Australian genus *Sarothrias* Grouvelle 1918 (Coleoptera, Jacobsoniidae). Tropical Zoology 3: 237–241. doi: 10.1080/03946975.1990.10539466

[B2] Dajoz R (1978) Une espèce nouvelle de l´Inde de genre *Sarothrias* Grouvelle (Coleopteres, Sarothriidae). Bulletin Mensuel Societe Linneenne Lyon 47: 322–324.

[B3] Háva J Löbl I (2005) A world catalogue of the family Jacobsoniidae (Coleoptera). Studies and Reports of District Museum Prague-East, Taxonomical Series 1, 1(1–2): 89–94.

[B4] Heller KM (1926) Fauna sumatrensis (Beitrag Nr. 29). Rhysodidae et Familia nova Jacobsoniidae (propre Rhysodidae? Col.). Supplementa Entomogogica 14: 126–128.

[B5] Lawrence JF Beutel RG Leschen RAB (2010) 5. Derodontiformia. Introduction, Phylogeny. In: Leschen RAB, Beutel RG, Lawrence JF (Volume eds) Coleoptera, beetles. Volume 2: Morphology and systematics (Elateroidea, Bostrichiformia, Cucujiformia partim). In: Kristensen NP Beutel RG (Eds) Handbook of zoology. A natural history of the phyla of the animal kingdom. Volume IV. Arthropoda: Insecta. Part 38 Walter de Gruyter, Berlin, New York, 179–180.

[B6] Lawrence JF Hastings AM Dallwitz MJ Paine TA Zurcher EJ (1999a) Beetle Larvae of the World: Descriptions, Illustrations, Identification, and Information Retrieval for Families and Subfamilies. CD-ROM, Version 1.1 for MS-Windows. CSIRO Publishing, Melbourne.

[B7] Lawrence JF Hastings AM Dallwitz MJ Paine TA Zurcher EJ (1999b) Beetles of the World: A Key and Information System for Families and Subfamilies. CD-ROM, Version 1.0 for MS-Windows. CSIRO Publishing, Melbourne.

[B8] Lawrence JF Leschen RAB (2010) 5.3 Jacobsoniidae Heller, 1926. In: Leschen RAB, Beutel RG, Lawrence JF (Volume eds) Coleoptera, beetles. Volume 2: Morphology and systematics (Elateroidea, Bostrichiformia, Cucujiformia partim). In: Kristensen NP Beutel RG (Eds) Handbook of zoology. A natural history of the phyla of the animal kingdom. Volume IV. Arthropoda: Insecta. Part 38 Walter de Gruyter, Berlin, New York, 190–195.

[B9] Lawrence JF Newton AFJ (1995) Families and subfamilies of Coleoptera (with selected genera, notes, references and data on family-group names). In: Pakaluk J Ślipiński SA (Eds) Biology, Phylogeny and Classification of Coleoptera: Papers Celebrating the 80th Birthday of Roy A. Crowson. Muzeum i Instytut Zoologii PAN, Warszawa, 779–1006.

[B10] Lawrence JF Ślipiński A Seago AE Thayer MK Newton AF Marvaldi AE (2011) Phylogeny of the Coleoptera based on morphological characters of adults and larvae. Annales Zoologici (Warszawa) 61(1): 1–217. doi: 10.3161/000345411X576725

[B11] Lin MY Yang XK (2012) Acanthocnemidae and Plastoceridae newly recorded from China (Coleoptera). Acta Zootaxonomica Sinica 37(2): 447–449, 8 figs.

[B12] Löbl I Burckhardt D (1988) Revision der Gattung *Sarothrias* mit Bemerkungen zur Familie Jacobsoniidae (Coleoptera). Stuttgarter Beiträge zur Naturkunde, Ser. A, 422: 1–23.

[B13] Pal TK (1998) A new species of *Sarothrias* Grouvelle from Northeast India. Doriana 7(307): 1–7.

[B14] Philips TK Ivie MA Giersch JJ (2002) Jacobsoniidae. In: Arnett RHJ Thomas MC Skelley PE Frank JH (Eds) American Beetles. Volume 2. Polyphaga: Scarabaeoidea through Curculionoidea. CRC Press LLC, Boca Raton, 219–220.

[B15] Poggi R (1991) Descrizione di una nuova specie papuana del genere *Sarothrias* Grouvelle (Col. Jacobsoniidae). Annali del Museo Civico diStoria Naturale “G. Doria“88: 677–683.

[B16] Ślipiński SA (1986) Description of two new species of *Sarothrias* Grouvelle (Coleoptera, Jacobsoniidae). Revue Suisse de Zoologie 93: 59–62.

[B17] Ślipiński SA Löbl I (1995) New species of *Sarothrias* (Coleoptera, Jacobsoniidae). Bulletin de la Société Entomologique Suisse 68: 49–53.

[B18] Zhang YW (1988) Coleoptera: Trogidae. In: Huang FS Wang PY Yin WY Yu PY Lee TS Yang CK Wang XJ (Eds) Insects of Mt. Namjagbarwa region of Xizang. Science Press, Beijing, 233–237.

